# Adapting despite *“walls coming down”*: Healthcare providers’ experiences of COVID-19 as an implosive adaptation

**DOI:** 10.1007/s40037-022-00716-w

**Published:** 2022-05-30

**Authors:** Sayra Cristancho, Emily Field, Taryn Taylor

**Affiliations:** 1 Department of Surgery, The University of Western Ontario, London, Canada; 2 Centre for Education Research & Innovation (CERI), The University of Western Ontario, London, Canada; 3 Department of Obstetrics and Gynecology, The University of Western Ontario, London, Canada

**Keywords:** Teams, Adaptation, COVID-19, Healthcare

## Abstract

**Introduction:**

The COVID-19 pandemic has been a daunting exercise in adaptation for healthcare providers. While we are beginning to learn about the challenges faced by teams during the COVID-19 pandemic, what remains underexplored are the strategies team members used to adapt to these challenges. The goal of this study is therefore to explore how healthcare providers navigated and adapted to on-the-ground challenges imposed by COVID-19.

**Methods:**

We interviewed 20 healthcare workers at various hospitals in Ontario, who provided care as part of clinical teams during the COVID-19 pandemic. Data were collected and analyzed following Constructivist Grounded Theory principles including iteration, constant comparison and theoretical sampling.

**Results:**

Participants’ accounts of their experiences revealed the process of ‘implosive adaptation’. The ‘reality check’, the ‘scramble’ and the ‘pivot’ comprised this process. The reality check described the triggers, the scramble detailed the challenges they went through and the pivot prescribed the shifting of mindset as they responded to challenges. These stages were iterative, rather than linear, with blurred boundaries.

**Discussion:**

According to our participants, not all adaptations have to be successful during a crisis. The language of reality check, scramble and pivot provides a framework for teams to talk about and make sense of their approaches to crisis, even beyond the COVID-19 pandemic.

**Supplementary Information:**

The online version of this article (10.1007/s40037-022-00716-w) contains supplementary material, which is available to authorized users.

## Introduction

The COVID-19 pandemic has been a daunting exercise in adaptation for healthcare providers who have had to dramatically change the way they do their work with little time to make sense of those changes. We are only beginning to learn about the challenges that the COVID-19 pandemic imposed on healthcare systems and healthcare providers [1–3]. However, what remains underexplored are the ways teams identified and adapted to the on-the-ground challenges of their daily work. COVID-19 will not be the last pandemic our society will endure; nor will pandemics be the most difficult hurdle faced by healthcare teams. We believe that understanding how team members made sense of, and responded to, the on-the-ground challenges imposed by this crisis carry important insights. And these insights will become critical not only for working in future pandemics, but also for working in situations that require high levels of adaptability.

COVID-19 has impacted healthcare teamwork in every hospital worldwide [3, 4]. Healthcare systems around the globe have continued to care for patients despite a shortage of hospital beds, staff and medical equipment [5], supply chain disruptions [6, 7], and limited laboratory capacity [8], among other challenges. While these constraints are a daily reality in resource-poor settings, such scarcity is unfamiliar to many providers in resource-rich countries. Faced with the unprecedented need to triage, ration and alter provision of care in unprecedented ways, healthcare providers suffered from burnout, anxiety and guilt [9, 10]. In this way, COVID-19 did not create one but many pandemics [11]. Not only did COVID-19 further expose troubling global health disparities, it also disrupted the everyday work of healthcare teams who were increasingly forced to do more with less.

Daily routines were profoundly disrupted, which required healthcare teams to adapt rapidly. They navigated operational and psychological challenges. For instance, some have documented the various changes to management strategies their teams adopted to prepare for the influx of patients; from expanding bed capacity to creating negative pressure rooms [2, 12, 13]. Others have documented the moral distress and fear healthcare providers experienced when caring for colleagues who tested positive or dealing with shortages of personal protective equipment [9]. While some of these challenges were more pressing in the early waves, as the pandemic has evolved, so have the types of challenges [14–16].

Novel challenges are sometimes difficult to talk about, leading to ineffective communication. And without effective communication, solutions may be inaccessible. Part of the issue may rest in lack of common language due to lack of experience with this type of crises. Research exploring teamwork in extreme contexts can provide some of that language. In a recent review of the science of teamwork, the authors summarized the dimensions of teamwork that may be impacted by COVID-19: collective efficacy, task vigilance, team trust, psychological safety, shared mental models and team conflict [17]. While useful to guide conversations, we still know little about how healthcare providers are making sense of the burden that COVID-19 has placed on healthcare teams. Empirical studies are beginning to be published [3]. These studies have mainly focused on teasing out the common issues that healthcare providers shared about their experiences with COVID-19. Our study aims to advance this knowledge by exploring how healthcare providers navigated and adapted to on-the-ground challenges imposed by COVID-19. The complex problems of our society will continue to test the resilience of healthcare systems. While healthcare systems may have been impacted differently across the globe, daily on-the-ground challenges may have emerged similarly for teams in the frontlines. It is therefore critical that we continue to build useful language that enables healthcare teams, regardless of context, to talk about and find ways to adapt within the current crisis and when facing any future, inevitable disruptions.

## Methods

Because we were interested in the process of adaptation, we utilized Constructivist Grounded Theory (CGT), which is well suited to understanding dynamic social processes and interactions [18].

We utilized both purposeful sampling and snowball sampling strategies [19]. In total we recruited 20 healthcare providers across multiple fields and disciplines—including, nursing, emergency medicine, intensive care, anesthesia and surgery—at various hospitals in Ontario, Canada, who provided care as part of clinical teams during the COVID-19 pandemic. Data collection occurred throughout the pandemic starting in April 2020 and concluding in April 2021. Participants were invited to participate in a single, 60-minute, audio-recorded semi-structured interview via Zoom with EF or SC. Participants were asked to share experiences where their teams had to navigate challenges imposed by COVID-19. Once specific situations were described by participants, they were also invited to share additional insights around questions such as: What were the surprising/unexpected issues during that situation? How did they evolve? How did your team navigate and adapt to those issues? How was teamwork impacted?

Data collection and analysis occurred simultaneously, as per CGT principles. This analytical process occurred in three progressively interpretive stages: initial, focused and theoretical.

### Initial coding

The first five transcripts were read line-by-line and coded by SC and EF using gerunds (action words ending in ‘ing’), and participants’ words (in vivo codes) to capture the meanings and actions described by participants. The intention of this first stage of analysis was to describe, rather than interpret, participants’ perspectives and experiences to ensure that preliminary findings were firmly ‘grounded’ in the data.

### Focused coding

Next, EF applied the initial coding framework to code five more interviews. EF, SC and TT met to consolidate the initial codes into preliminary themes that were used to focus code five more transcripts.

### Theoretical coding

A coding framework (see Appendix in the Electronic Supplementary Material) was finalized by EF, TT and SC and used to develop a preliminary conceptual model. This model was presented to five additional participants during return-of-finding interviews. These interviews were used to gather insights into whether our preliminary findings resonated and to refine the definitions in our coding framework. The final coding framework was then applied to re-code the entire dataset and consolidate our conceptual findings. The steps followed in this analytical process ensured the rigor of the analysis by considering resonance, trustworthiness and transferability [20]. All study procedures received Ethics Approval through Western Research Ethics Manager, project ID #113589 and Lawson ReDa #6356.

SC, EF and TT are all qualitative research experts. In addition, SC’s research program focuses on understanding professional teams’ responses to unexpected events in high-stakes industries, such as healthcare. Her expertise allowed the research team to anchor the focus of the study on participants’ experiences with working in teams. EF is a feminist scholar who adds a critical perspective to the analysis. She helped the research team unpack the social and political nuances of participants’ experiences. TT is a physician who remained on the frontlines throughout the pandemic. She brought her personal experience to help the research team contextualize the findings and their implications for healthcare providers on-the-ground. This research team met frequently throughout this process to combine their perspectives and deepen the analysis of the data as well as examine how themes evolved.

## Results

The COVID-19 pandemic forced all healthcare workers to adapt to a greater or lesser extent. The participants in this study had to adapt their day-to-day work, but unlike New York or Italy, did not have an onslaught of patients during the first or second wave. However, as they entered the third wave, *“there has been a tectonic shift” *(p20). For most, adaptation came with a sense of urgency and “*we adapted, but it was an implosion, it was not a successful adaptation”* (p14).

**Fig. 1:**
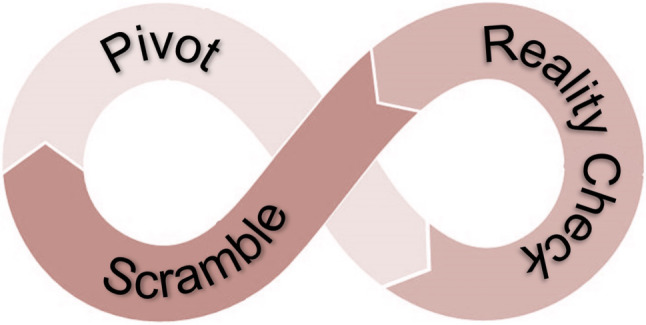
The process of implosive adaptation

As we attempted to tease out this process of **implosive adaptation**—i.e., having to adapt amidst *“walls coming down”* (p19)—we identified three overlapping stages that healthcare providers worked through with their teams to navigate COVID-19 (Fig. 1). First, rapid changes in the usual way of doing work sparked a **reality check** for team members. They described the triggering moments that made them realize that things could deteriorate rapidly. Once the situation became critical, team members faced multiple challenges that forced them to **scramble**. Most challenges involved working at odds with traditional values. Team members wrestled with the reality that preserving autonomy and hierarchy clashed with efficiency and productivity. As they began to realize what did and did not work early in the pandemic, team members recognized the need to shift their mindset to be able to **pivot** and move forward. For most participants, these three stages *“resonated with the cycle of change *[they]* went through”* (p19).

The rest of this section will provide a narrative illustration of the key dimensions of each stage. These narratives have been composed from participants’ quotes [21] in order to preserve the accuracy while offering diversity of participants’ experiences.

### The reality check

A “reality check” occurred for participants when it became apparent that COVID-19 was a global pandemic that was likely going to walk through the doors of their hospitals. This shift in their reality created moments of disruption that were a catalyst for adaptation. Participants described it in several ways.

For many it felt like “*every single piece of the puzzle was shifting from masking to where you could travel in the hospital to how people needed to be isolated to who is taking care of patients”* (p2). They lost the sense of certainty about what to do on a day-to-day basis. Coupled with shortages and conflicting understandings of personal protective equipment, the clinical work environment completely shifted. This was in part because of the acuity of the crisis, but mostly “*because of the changing processes and changing rules. So, you’re never quite sure whether you’re doing the right thing” *(p5).

While COVID did not overwhelm the local system during the first wave, team members did not escape the anxiety of remaining alert. Many of them talked about the calm before the storm and described it as *“being backstage *[as]* more anxiety provoking than being on stage”* (p3). And missing the sense of certainty added to the anxiety because *“none of us were naturally trained to think about how to prepare for a pandemic”* (p9).

Those feelings brought the realization that expertise became a commodity in high demand. For some, it was *“so pathetic because we really have no expertise but we’re the experts. And so, it was just this terrifying reality” *(p9). After the acuity of the first wave receded, participants understood that being experts without expertise not only applied to treating COVID patients. For patients with chronic conditions, they began asking questions they were not used to asking because *“COVID itself added that layer of complexity that made you second guess”* (p17).

In addition to the rapid changes in the usual way of doing work, the reality check brought out the evolving fears providers experienced over the course of the pandemic:

*Talking to colleagues in New York, they told us, you’re going to know somebody, whether it’s a colleague or a family member or yourself, who is going to die from this. So, there was actual fear about safety for yourself, bringing the virus home and giving it to loved ones. And there was real concern from the provision of care about what’s going to happen if, say, doctors and nurses get sick, and we don’t have the staffing because of that? Now with vaccines out and this wave, it’s a different fear, we’re not worried about running out of equipment, we’re not worried about personal safety, it’s the numbers of beds and access to ICU and the effect on non-COVID patients and their access to care *(p20)*.*

In dealing with these reality checks, team members faced multiple challenges that forced them to scramble as they tried to care for others and themselves.

### The scramble

Early in the pandemic, participants agreed with the strategies re-deploying people to different roles or tasks. The way units were staffed changed, *“we took the three or four younger, healthier nephrologists, and say, you’re now our ICU nephrologist … and that was a big win”* (p11). While redeployment was viewed as an effective strategy by most, it was difficult for many like *“these operating room nurses *[who]* all of a sudden some are in Emerg, some are in ICU, some are in the COVID assessment unit” *(p3)*,* who found themselves working outside their usual scope of practice with little time to mentally adapt to this change. As time passed, people realized that it was reasonable to work outside their scope, and even *“were thankful for those that are willing to bend their scope”* (p13).

Working outside the scope brought benefits but also challenges. For instance, a major scramble for participants in our study related to mitigation strategies that had unintended consequences, such as the formation of special COVID airway teams. The scientific rationale was justified based on previous experience with *“SARS‑1 in the ‘90s *[which]* found that you want the most skilled people because you want it to go smoothly”* (p18). Therefore, the assumption was made that anesthesiologists would be the ideal specialists to compose these teams. However, for other specialists who were routinely responsible for airway management in emergencies, it felt like *“the things that we have within our scope being done by others”* (p14), but also being done with a different sense of what’s important in the moment. It was described as *“this clash of cultures, like Emergency, like ICU, where moving fast, adapting quickly is just part of the culture. But then the strategies of bringing in people from other specialities that had a different mindset created a whole sort of issues”* (p19). The perception that unfamiliar team members brought a different mindset was particularly frustrating and unwelcomed. This led teams to implement informal work around strategies during the first wave. For instance, reinforcing role separation by keeping *“the trauma team function[ing] autonomously and independently, and treat[ing] the intubation team, for better or for worse, as technicians, not as a consultative service”* (p11). The airway team strategy looked good on paper, but its potential was not fully realized. For some the problem was *“people kind of being dropped in”, *rather than *“somebody who was going to be a part of our team”*. If the latter had been a possibility,* “I think we could meet in the middle”, *as some participants indicated. Specially during the first wave, it felt like *“many of these processes *[were]* just dumped in”* (p15).

The scramble of having to incorporate unfamiliar team members created strong dissonance that prompted an explicit reflection after the first wave. This reflection involved thinking about *“how to integrate one *[or] *more groups into our team” (p19). *As healthcare providers became more used to managing COVID patients, the reflection resulted in explicit changes to these teams. As one intensivist described it, *“it kind of went from being very awkward and almost shunned and pushed away initially to us getting more comfortable. By any means it’s not perfect but it fits in a lot better with trauma care and ICU care”* (p19).

For other healthcare teams, the scramble involved adapting to the loss of team members and the loss of wider support from other specialties. Participants working directly with COVID patients outside of the ICU described being viewed as *“lepers”* (p6) by colleagues in other specialties resulting in *“a huge dichotomy between those who are actually doing COVID and those who aren’t”* (p13). This dichotomy left healthcare providers having to either appeal to their colleagues to *“please, just do this”* (p2) or expand their usual scope of practice to *“put that thing in yourself or do that procedure yourself”* (p6). In other instances, many felt the loss of support when they *“looked at that *[redeployment]* survey”* and realized that *“they’re clearly not interested in sending anyone to Emerg. This was an ICU questionnaire”* (p6). The scramble for these teams was not only to adapt *“their roles to work for the greater good”* (p13) but to maintain their cohesiveness as *“people had to *[be]* pulled together to support each other”* (p4). This involved covering each other’s shifts, offering to be the *“COVID nurse”* (p7) when your fellow colleagues were concerned, and working to improve team morale.

Overall, we found that teams scrambled the most when incorporating unfamiliar team members or losing the support of specialities, which forced them to work outside their usual scope. For the former, teams had to adapt to mitigation strategies that took experts out of one context (airway management in the operating room) and put them in a different context (airway management in the emergency room) while expecting them to immediately adapt to, and be welcomed into, that context. For the latter, teams had to adapt to lack of support, which threatened the way they carried out work and the team’s cohesiveness. As team members began to realize what worked and what didn’t work early, they also realized the need for a pivot in mindset to find their way out of the scramble.

### The pivot

As the pivot entails a shift in mindset, healthcare providers in our study reflected on the many ways in which their perspectives shifted. We found that one of the major pivots in mindset occurred in relation to the idea that *“there is no emergency in a pandemic”* (p20). Most participants reflected that the biggest change was realizing that even though *“it’s really hard to have to stop, [you must] accept the greater good of the rest of the team by putting your PPE on because you cannot be lost.”* (p17). According to another participant,

*We will lose some patients because of that, but you have to protect the staff. Losing a patient from a cardiac arrest during COVID-19 is terrible. Losing two nurses because they got exposed, is a disaster. So, that was a weird thing for everybody to get their head around* (p15).

Also, as redeployment became more normalized, team members accepted that they could* “likely be redeployed anywhere. I haven’t worked in Emerg for a while but, should they have needed me there I would have been able to go there” *(p3). The learning that came from embracing the idea that making people interchangeable was reasonable, gave team members the courage to *“simply sa[y] plug me in where you need me” *(p3)*.*

Finally, participants illustrated how the pivot in mindset “*has felt like a whole exercise in dissonance” *(p18). Participants referred to dissonance as the inconsistencies they encountered between the principles that they were supposed to espouse and the actions they found themselves and others enacting. Dissonance revealed in various forms:

*I have a professional duty but when I look at the behaviour of some in the general public; that creates dissonance. I know I am an independent professional but there are all these rules being imposed on me; that creates dissonance. I have gathered scientific knowledge to support why we should be doing something, but other experts disagree with me; that creates dissonance *(p18).

For most, the pivot in mindset that allowed them to manage this dissonance hinged in whether *“the different specialties have had an opportunity to develop a culture of responding to challenges”* (p14).

## Discussion

The reality check, the scramble, and the pivot comprised **the process of implosive adaptation **that we found most of our participants engaging with. As they navigated this process of implosive adaptation, they realized that as multiple reality checks were thrown at them, there was no time to dwell on them. They quickly found themselves dealing with the challenges of the scramble. And as long as the feeling of scrambling was present—with more waves appearing—the pivot persisted. While described in three separate stages for clarity’s sake, these stages were iterative, rather than linear, with blurred boundaries (Fig. 1). Nonetheless, as our participants reflected during our return-of-findings interviews, the separation of the stages afforded a language that they found useful to reflect on and share their experiences with others.

As it has already been reported, working outside scope was a mitigation strategy that most hospitals deemed necessary [1, 2, 4, 22]. In our study, participants indicated that working outside scope happened in one of three ways: by switching people around—commonly referred to as redeployment, by doing tasks outside or beyond their training level or experience, and by having to allow others outside their teams do tasks that were normally part of their team’s scope. Having to work outside scope because of lack of support or having to grant their scope to others outside their teams were particularly controversial in our study. For instance, while the creation of the airway teams was justified by lessons learned during the SARS epidemic, its implementation was fraught with challenges. That not all adaptations have to be successful during a crisis was the major insight gained by our participants, which should prompt reflection about dealing with future challenges.

As crises cannot always be predicted or controlled, teams must be prepared to look for and find expertise in places they might not have anticipated before. In the Global North, healthcare providers are accustomed to accessing expert guidelines that dictate how to practice safely. However, during crises like COVID-19, safety at all costs might not be possible. A compromise must occur between striving for safety and leaving room for thinking creatively [23]. Therefore, as the lines between individual specialties begin to blur, teams should allow for the emergence of new experts—those whose out-of-the box thinking allows teams to come up with context-specific solutions amid resource limitations [24]. Such expertise does not reside on positions or roles, hence the need to promote conversations among team members that help flattening hierarchies and fostering a productive environment for exchanging ideas. The language of the implosive adaptation model proposed in this study could serve as a framework for teams to guide these conversations.

COVID-19 not only imposed operational challenges to healthcare teams, it also permeated into their personal lives. The psychological impact of COVID-19 served as preamble of the next impending crisis in healthcare: dwindling human resources due to provider burnout. Healthcare provider burnout was a problem pre-pandemic [25]. However, the pandemic has amplified issues such as moral injury [26] and compassion fatigue, leading to a mass exodus of healthcare workers across all healthcare sectors [27]. More health workers will be required. But the discussion should not focus on numbers alone [28]. COVID-19 exacerbated the discourse of shortages which should force us to rethink training, deployment and management of the health workforce. It will require a paradigm shift where future providers are trained to reflect on the implosive nature of crises that require them to be prepared to perform continuous reality checks, and to capitalize on their scrambles so that they can quickly pivot their solutions.

While this study is not in the position of suggesting rectifications to the architecture of global health partnerships and institutions, it contributes to a call for in-depth qualitative research to evidence the on-the-ground experiences and impacts of the pandemic [29]. Overall, this study offers a window into the limitations and blind spots of one-size-fits-all pandemic responses. Furthermore, it provides a language to help healthcare teams reflect on what the various phases of a crisis might look like and how they can drive conversations among team members to find a way forward with whatever resources are available to them.

### Limitations

The findings presented here are bounded by research design decisions that draw attention to certain aspects and deflect attention from others. For instance, our decision to sample team members from a variety of specialties allowed us to identify the patterns that informed the articulation of the three stages of the implosive adaptation process but prevented us from exploring in detail how specialties navigated each stage of the process. Similarly, our decision to sample in a Global North context allowed us to consider the experiences of this crisis as lived by healthcare providers who did not have to bear the political pressures of, for instance, vaccine nationalism, as they were experienced by providers in the Global South. Future work should investigate the cultural features of different clinical, social and political environments that influence a team’s response to challenges in the geographical context they occupy. Similarly, even though interpersonal dynamics were not the focus of this study, some participants shared stories of animosity built within and across teams, suggesting that future work should attend to the impact of affect in how teams worked together. Finally, since COVID-19 has been a dynamic pandemic, the lessons articulated here are contextual and likely still in flux as the situation continues to unfold.

## Conclusion

In this paper, we have described the process of implosive adaptation that healthcare team members went through as they navigated the challenges imposed by COVID-19. While simplistic in appearance, the language of reality check, scramble and pivot provides a framework for teams to talk about and make sense of their approaches to crisis, even beyond the COVID-19.

## Supplementary Information

Categories of data analysis with sample of quotes.
