# Supplementary material for: Adapting despite “walls coming down”: Healthcare providers’ experiences of COVID-19 as an implosive adaptation
Source: Perspect Med Educ. 2022 May 30;11(4):213–9. doi: 10.1007/s40037-022-00716-w (PMC9150045; doi:10.1007/s40037-022-00716-w)
Supplement: Supplementary file 1 — Categories of data analysis with sample of quotes. [file 40037_2022_716_MOESM1_ESM.docx]

**Appendix**

**ATA COVID CODEBOOK**

**Areas of Adaptation/Preparation**

- Are we prepared?
- Human Resources
- Patient Flow/Triage/Testing
- Policies/Procedures/Processes
- Space
- Mindset

**Adaptation Timeline**

- Evolving over time
- Calm before the storm
- The new normal
- Second wave
- Key Stories

**Experts without Expertise**

- What we think we know vs what we actually know
- Crowd sourcing

**Expanding**

- Redeployment
- New team members
- Expanding Scope/Shifting Role
- Stepping up

**Managing Information Overload**

- Information overload
- Strategies/Streamlining

**Impacts**

- Accountability
- Emotional Impacts on Individuals
- Financial
- Impacts on Patients
- Manifestations of Anxiety
- Impact on Teams (Pos and Neg)

**Problems of Bureaucracy**

- Priorities
- Speed of Response

**Public Discourse**

**PPE**

- Competing Understandings
- Fears/Shortages

**Safety**

- HCP First
- Tensions

**Simulation**

- Debrief
- Importance
- Protected Code Blue

**Lessons Learned**

- What went well
- What went wrong
- Institutional memory/legacy
